# Minimal Interference from Possessor Phrases in the Production of Subject-Verb Agreement

**DOI:** 10.3389/fpsyg.2016.00548

**Published:** 2016-05-02

**Authors:** Janet L. Nicol, Andrew Barss, Jason E. Barker

**Affiliations:** ^1^Department of Linguistics, Program in Cognitive Science, University of ArizonaTucson, AZ, USA; ^2^Department of Psychology, Program in Cognitive Science, University of ArizonaTucson, AZ, USA; ^3^Formerly Affiliated with Department of Psychology, Program in Cognitive Science, University of ArizonaTucson, AZ, USA

**Keywords:** subject-verb agreement, possessive, possessor, genitive, production error, attraction error, case marking, semantic integration

## Abstract

We explore the language production process by eliciting subject-verb agreement errors. Participants were asked to create complete sentences from sentence beginnings such as *The elf's/elves' house with the tiny window/windows* and *The statue in the elf's/elves' gardens.* These are subject noun phrases containing a head noun and controller of agreement (*statue*) and two nonheads, a “local noun” (*window(s)/garden(s)*), and a possessor noun (*elf's/elves'*). Past research has shown that a plural nonhead noun (an “attractor”) within a subject noun phrase triggers the production of verb agreement errors, and further, that the nearer the attractor to the head noun, the greater the interference. This effect can be interpreted in terms of relative hierarchical distance from the head noun, or via a processing window account, which claims that during production, there is a window in which the head and modifying material may be co-active, and an attractor must be active at the same time as the head to give rise to errors. Using possessors attached at different heights within the same window, we are able to empirically distinguish these accounts. Possessors also allow us to explore two additional issues. First, case marking of local nouns has been shown to reduce agreement errors in languages with “rich” inflectional systems, and we explore whether English speakers attend to case. Secondly, formal syntactic analyses differ regarding the structural position of the possessive marker, and we distinguish them empirically with the relative magnitude of errors produced by possessors and local nouns. Our results show that, across the board, plural possessors are significantly less disruptive to the agreement process than plural local nouns. Proximity to the head noun matters: a possessor directly modifying the head noun induce a significant number of errors, but a possessor within a modifying prepositional phrase did not, though the local noun did. These findings suggest that proximity to a head noun is independent of a “processing window” effect. They also support a noun phrase-internal, case-like analysis of the structural position of the possessive ending and show that even speakers of inflectionally impoverished languages like English are sensitive to morphophonological case-like marking.

## Introduction

When speakers produce language, they need to map the elements of a to-be-conveyed proposition onto an appropriate sentence structure, and keep track of these assignments as the utterance is produced. This process is relatively straight-forward for simple sentences like *The key is shiny*, but becomes more challenging when additional information needs to be encoded. For example, in *The key to the cabinets is shiny*, the subject of the verb is the phrase *the key to the cabinets*; however within this phrase, *key* is the head, and *cabinets* is part of a modifying phrase, and the head noun must be selected as the controller of verb agreement. Speakers need to keep this distinction in mind if they are to produce sensible sentences: for the most part, the head noun is the thing the sentence is about, the main element of the predicate's argument (the key, not the cabinets, is what is shiny), and the element a verb may need to agree with. Occasionally, the process goes awry, and a subject-verb agreement error is the result. Studying the variables that affect the incidence of such errors illuminates the language production process.

Subject-verb agreement errors occur with some regularity in both spoken and written language (Jespersen, 1913/1961; Visser, 1963; Quirk et al., 1985; Bock and Miller, 1991). In a seminal paper, Bock and Miller (1991) elicited errors in the laboratory by presenting participants with sentence beginnings, or *preambles*, and asking them to repeat these and create a sentence ending that included a verb. The results showed that agreement errors arise when a singular head is modified by a prepositional phrase containing a plural noun (typically called the *local noun*, or when it is plural, the *attractor*); e.g., *The key to the cabinets were shiny*. The error is not simply due to participants' forgetting the head and implementing agreement “locally” between the attractor and the verb because *The keys to the cabinet* does not elicit errors at the same rate. One explanation for the difference is that the singular is seen as the default: a plural is derived from the singular by the addition of a marked feature, and this plural feature has an autonomy that allows it to intrude on the number specification of a verb. Since that initial study, a great deal of research has explored the kinds of variables that influence the production of agreement errors, and these have led to a refinement and elaboration of syntactic encoding operations in language production.

The focus of this paper is the production of subject-verb agreement errors in English sentences containing a complex subject noun-phrase that includes a singular head noun and a local noun^1^, but also a possessor phrase bearing the possessive marker [“s/”], as in (1) and (2). Our experiments examine possessors in two positions: modifying the head noun, as in (1), and modifying a local noun, as in (2).

(1) The women's subscription to the newsletter…
(2) The subscription to the women's newsletter…

The effects of possessors have, until now, been unexplored. In addition to expanding the range of constructions examined in this experimental paradigm, they permit us to explore two issues: (a) the nature of the structural effects that have been argued to influence the presence and magnitude of errors; and (b) the role of the possessive ending as a potential cue to non-subjecthood in potentially reducing errors, akin to the role that overt case marking has been found to play in several languages.

These two issues, and predictions for our experiments, are detailed below.

### Proximity effects

Previous research has shown that an attractor that appears within a modifier that is adjacent to the head noun triggers more errors than one that is located more distantly from the head noun. Several accounts have been offered for this difference, which make contrasting predictions with respect to the behavior of the two types of possessors in (1)–(2).

First, consider some of the empirical findings. Bock and Cutting (1992) found that an attractor within a prepositional phrase (PP) modifier (e.g., 3 and 5 below) elicits significantly more agreement errors than a plural attractor within a clausal modifier (e.g., 4 and 6), and further, that a plural attractor within a relative clause modifier (e.g., 4) elicits more errors than one in a complement clause (e.g., 6).

(3) The editor of the history books
(4) The editor who rejected the books
(5) The dream about the castles
(6) The dream that Anne inherited the castles

**Figure 1:**
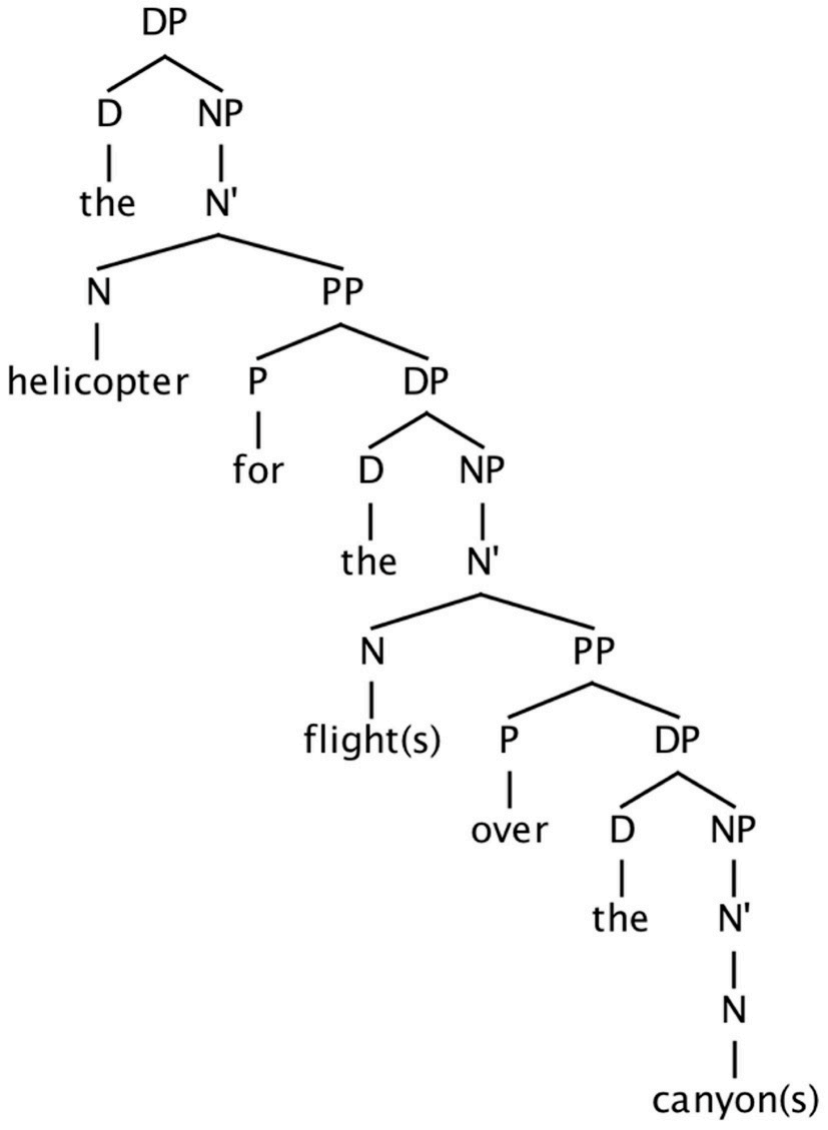
**Approximate structure for examples (7) and (8), showing relative embedding of two PP modifiers**.

Further proximity effects are presented by Franck et al. (2002), who examined contrasts like the following, in which a plural attractor appears inside one of two PP modifiers with different syntactic attachment heights (see Figure 1):

(7) The helicopter for the flights over the canyon
(8) The helicopter for the flight over the canyons

A comparison of error rates associated with these sentence types revealed a substantial difference, with the latter eliciting very few errors.

Bock and Cutting (1992) argued that the difference in error rates between (6) and (3–5) was due to the extent to which the head noun and attractor are co-active, and that because a complement clause contains its own subject and predicate, its contents are insulated, in a sense, from the head, making an attractor less likely to be co-active with the head noun. An attractor within a PP or relative clause modifier is not insulated in this way. A variant of the co-activation view is that of Nicol (1995), who proposes that the verb-valuing operation must occur within the limited timeframe in which the subject noun phrase is active. A limited processing window exists, and only noun phrases that are co-active with the head noun (i.e., within the same processing window) will produce errors. This account extends to the complement-clause modifier vs. relative-clause/PP modifier contrast in (3–6), as well as the stacked PP modifiers in (7) and (8). The first modifier noun phrase down [e.g., *flights* in (7)] will be more likely than the deepest modifier (e.g., *canyons*) to be within this processing window, and therefore will be more likely to cause an agreement error. (A similar argument was reiterated by Gillespie and Pearlmutter, 2011).

Note that the processing window hypothesis treats the much lower rate of errors in (6) and (8) as a type of threshold effect: the attractors in those cases lie outside a processing window which includes the head noun and the more local plural attractor in (5) and (7). An attractor is either within the window, in which case it will potentially cause errors, or outside it, in which case error rates will be low. And the experimental results suggest that the processing window including the head noun extends rightward to encompass the first noun phrase, and not the second.

An alternative view is presented by Vigliocco and Nicol (1998). They attributed the difference to *relative* structural proximity of the head noun and attractor: the closer the attractor is to the head noun, the more likely it is to produce an error. In (5) and (7), the plural attractor is closer to the head noun (in terms of hierarchical distance, i.e., nodes separating the two) than the plural attractor is in (6) and (8), and thus more likely to cause errors.

Returning to our possessor phrases in (1) and (2), the two accounts make differing predictions with respect to expected error rates. Note that the possessors are embedded to different extents within the structure of the subject noun phrase. The head-noun-modifying possessor in (1) is more shallowly embedded than the local-noun-modifying possessor in (2), and closer to the head noun. On the relative structural proximity account, head-noun-modifying possessors should produce more errors than the local-noun-modifying possessors. The processing window analysis makes a different prediction: both possessors should lie within the processing window that includes the first PP modifier, and so both types of possessor should produce an equal number of errors. Our experiments compare error rates for the two possessor positions, allowing us to empirically distinguish the two accounts.

### The possessor ending and case marking

A number of studies have examined the effect of case marking of a local noun on attraction errors, and reported that overt case—case marking that is phonologically realized—acts to dampen errors. Case is variation in the form of a noun or determiner that depends on its grammatical function, e.g., subject, object, indirect object, oblique, etc., and is largely redundant with structural information. Yet the additional phonological marking appears to help speakers keep straight which noun is the agreement controller.

Local nouns inflected for case are less likely to produce errors when that case is unambiguously non-nominative (i.e., incompatible with the local noun being the head of a subject noun phrase) relative to local nouns that are either unmarked for case or bear case that could be nominative (i.e., a case marker that is ambiguous between nominative and non-nominative). The logic of this is clear: subject head nouns are typically nominative (either explicitly or covertly marked as such), and a local noun is less likely to become confused with this controller when its morpho-phonology is incompatible with subjecthood. Studies showing this effect include Nicol and Wilson (1999) and Lorimor et al. (2008) for Russian, Hartsuiker et al. (2001) for Dutch, Badecker and Kuminiak (2007) for Slovak, and Nicol and Antón-Méndez (2009) for English.

In the one study conducted on English, Nicol and Antón-Méndez (2009) created English preambles containing as the local noun either a non-casemarked full noun phrase or a case-inflected pronoun. Comparing e.g., *The bill from the accountants…* and *The bill from them…*, they found a significant reduction in the number of agreement errors associated with the case-marked condition. (The rate of agreement errors following the plural pronouns was about 6%, compared with 15% following full noun phrases which were not explicitly case-marked; this reduction in error rate by more than half mirrors that in several of the aforementioned studies).

We note that although case can be manifest somewhat differently in different languages, it always appears internal to the noun phrase^2^, a point relevant to our next prediction.

There is a puzzle presented by the prepositional phrase modifier cases that have been extensively studied in English, e.g., *the key to the cabinet(s)*, and which robustly produce attraction errors. Although neither the noun nor the determiner in *the cabinets* is casemarked, the presence of the preposition to the left of the determiner has a similar non-subjecthood signaling function: a noun phrase immediately preceded by a preposition is never the subject. Cross-linguistically, there is a close affiliation of case and preposition use. It is something of a puzzle, then, that the presence of case marking dampens agreement errors in a way that the occurrence of a preposition does not. A formal way to resolve this puzzle is to distinguish case marking from prepositions by observing that only the prepositions lie outside the noun phrase, and to conjecture that *only information internal to the noun phrase itself* is capable of acting as a non-subject cue strong enough to significantly reduce agreement errors.

This conjecture is relevant to formal syntactic treatments of the possessive. In the next section we review syntactic analyses of the possessive ending, and note that there is controversy as to whether the ending is part of the possessor phrase structurally (which would put it on a par with case inflection), or a structurally separate phrase-structure head, which would make it more like the noun-phrase-external prepositions which do not dampen agreement errors to the extent that casemarkers do. Exploring errors triggered by possessors offers a way to experimentally distinguish these formal analyses: an overall lower rate of errors with possessors would provide support for the noun phrase-internal view, and an error rate comparable to that with prepositional phrase modifiers would provide support for the noun phrase-external analysis of the possessor ending.

### The phrase structure of english possessives

**Figure 2:**
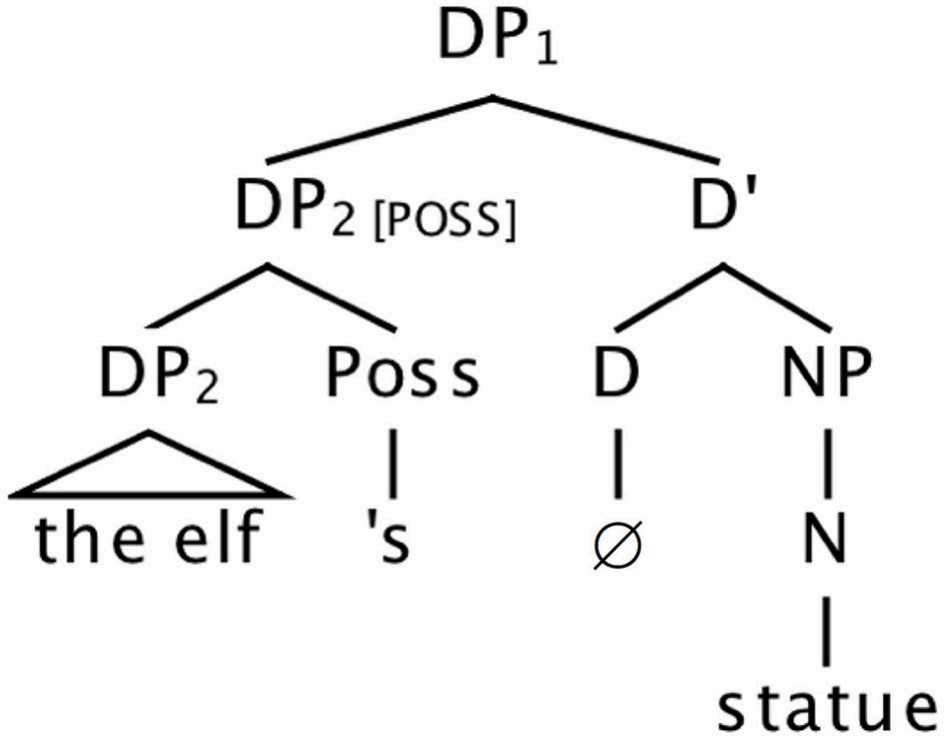
**Structure for ***the elf's statue***, with possessive ending analyzed as noun phrase-final syntactic clitic**.

**Figure 3:**
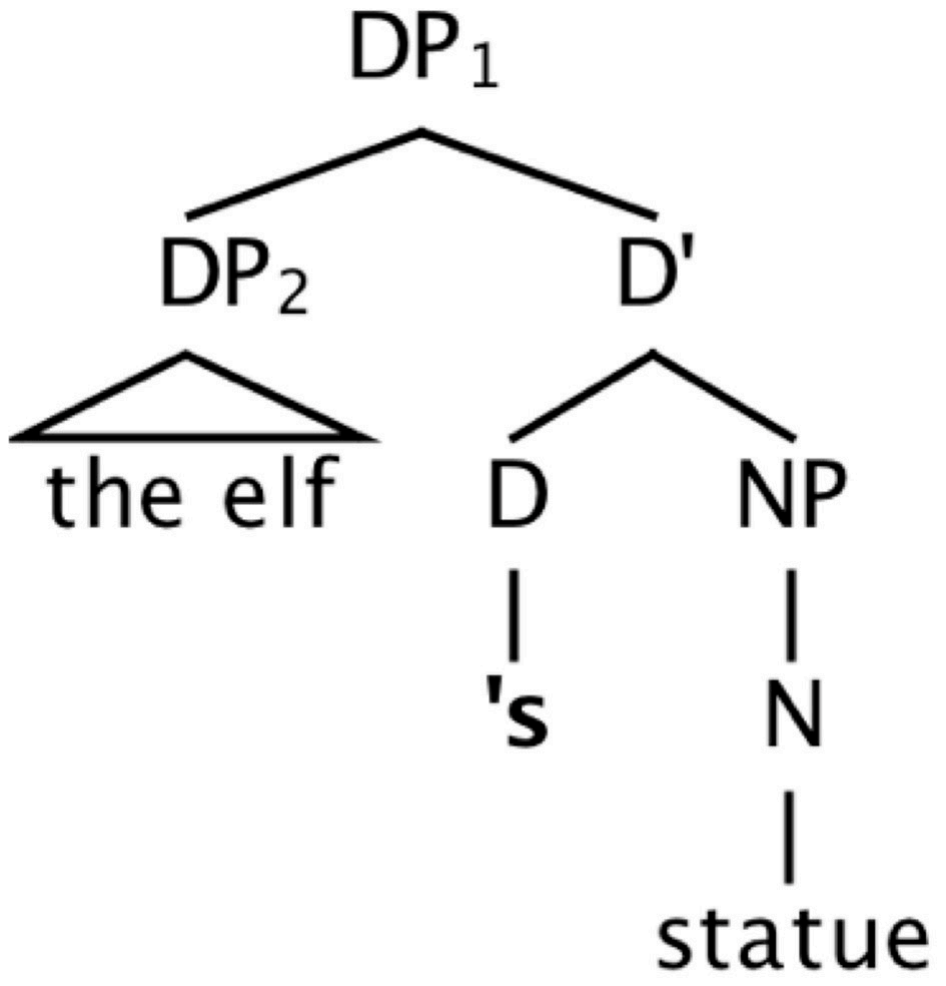
**Structure for ***the elf's statue***, with possessive ending as a syntactic determiner head, external to the noun phrase/DP**.

The possessive ending [“s/”] and the possessor phrase that it attaches to have received two distinct types of analysis in formal syntax. The possessor phrase itself occurs as the Specifier of a determiner phrase (DP). On the first type of analysis (Abney, 1987; Zwicky, 1987; Barker, 1991) the possessive ending is analyzed as a phrase-final affix, attached at the right edge of the possessor, as in Figure 2. We refer to this analysis as the *noun phrase-internal view* of the possessive ending, since it is both syntactically and morpho-phonologically part of the possessor noun phrase. On this account, the determiner of the overall possessive construction is null. On the second account, the possessive ending is analyzed as a syntactically autonomous determiner, as in Figure 3, which then phonologically encliticizes onto the possessor phrase (Abney, 1987; Delsing, 1998; Carnie, 2013). We refer to this as the *noun phrase-external view* of the possessor ending.

What is important for our discussion below about the latter analysis is that the ending is external to the noun phrase syntactically, occurring in a different region of the phrase structure. In this regard it is much like a preposition—occurring adjacent to, but not as a part of, the noun phrase itself. Since attractors in prepositional-phrase modifiers robustly elicit errors, the preposition—perhaps due to this structural separation from the noun phrase—apparently does not act as a strong cue for non-subjecthood in the way that noun phrase-internal case marking does in the studies discussed in the previous section showing a dampening effect of case marking.

**Figure 4:**
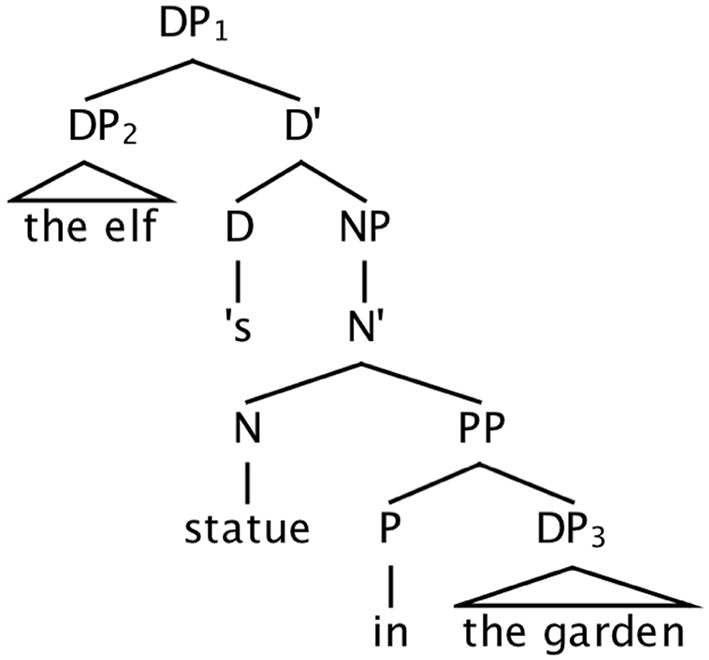
**Structure for ***the elf's statue in the garden***, showing possessive ending and preposition as DP-adjacent and DP-external heads**.

This pair of contrasting syntactic analyses of the possessor ending leads to differing predictions about the effect that ending will have on agreement errors. If possessors are robust attractors, this will be consistent with the *noun phrase-external* syntactic analysis (Figure 3) of the possessor ending, which treats the ending as a syntactically autonomous head, much like a preposition (see Figure 4). On the other hand, if possessors are weak attractors, this would be consistent with the analysis of the possessor ending that assimilates it to the class of *noun phrase-internal* case morphology (Figure 2).

In our materials, possessors either occur to the left of the head noun, as (9a) (Experiments 1 and 2), or to the right of the head noun (and to the left of the local nouns) when modifying the local noun, as in (9b) (Experiment 3).

(9)
  - The elves' statue in the garden
  - The statue in the elves' garden

Before turning to the experiments, we summarize the two sets of predictions we have presented. As noted above, the two possessor positions contrast in proximity to the head noun. Possessors of type (9a) are expected to have a higher error rate due to their greater proximity to the head noun, all other factors being equal, if the relative hierarchical proximity hypothesis (Vigliocco and Nicol, 1998) is the correct account of the locality effects seen in attraction errors. By contrast, the two possessor types should induce equal numbers of errors if the processing window hypothesis (Bock and Cutting, 1992; Nicol, 1995) is correct. And overall error rate for both types of possessors (compared to PP-contained local nouns) will reveal whether the possessor ending functions like noun phrase-internal case information in dampening errors, or a noun phrase-external preposition in not having such an effect.

## Experiment 1—auditorily-presented preambles, possessor modifies head

The purpose of the first experiment was to examine whether a plural possessor phrase modifying the head noun (type 9a) causes interference in the agreement process. For this first experiment, auditory presentation of preambles was chosen, in line with the majority of experiments using the usual paradigm of providing subjects with preambles to turn into complete sentences.

### Method

#### Participants

Forty-four native English-speakers participated in this experiment. Here, and in the studies described below: All were undergraduates at the University of Arizona who received course credit for their participation. They were native English speakers 18 years of age or older. All provided written consent to participate in these experiments, which had received prior approval by the University of Arizona Human Subjects Protection Program.

#### Materials

Because the stimuli were to be presented auditorily, we needed to ensure that the possessor was unambiguously singular or plural. This meant that we could not use possessors such as *girl's* and *girls'*, because these are homophonous. Therefore, the possessor in our experimental preambles was always a noun with an irregular plural form. The set of possessors included items such as *woman, child, person, housewife, midwife, wolf, thief, elf*.

Twenty quadruplets were such as those in (10) were created. (Here and throughout, preamble types are coded as follows: “s” = singular, “p” = plural, uppercase = head noun. Within the preamble examples, the head noun is underlined and plural nouns are boldfaced).

(10)
  - sSs The elf's house with the tiny window…
  - sSp The elf's house with the tiny **windows**…
  - pSs The **elves'** house with the tiny window…
  - pSp The **elves'** house with the tiny **windows**…

Each member of the quadruplet contained a singular head that was preceded by a possessor noun that was singular or plural, and followed by a prepositional phrase modifier containing a singular or plural noun. These were counterbalanced across four presentation lists such that a given participant was presented with only one member of a quadruplet (the full set of experimental items for this and subsequent experiments appear in the Supplementary Material). Each list also included 16 plural-head filler preambles which contained a singular possessor that modified either the head or the (singular or plural) noun within a PP modifier. In addition, there were 64 preambles that were the focus of a separate experiment. These contained a head noun followed by a PP and relative clause modifier; each of the three nouns was singular in half the items and plural in the other half. Finally, there were eight fillers that contained a head noun followed by a PP modifier; each of the two nouns was singular in half of the items and plural in the other half. The preambles were arranged in a fixed pseudorandom order (the same order for each list), and preceded by four practice items. The preambles were recorded by a female speaker.

#### Procedure

Participants were tested individually in a small test room. Preambles were presented auditorily over headphones. Participants were instructed to repeat each preamble and form a sensible completion. All utterances were recorded for transcription purposes.

#### Scoring

Transcribed sentences were scored using the following response categories: (a) Correct Inflected (the preamble was repeated correctly and the correct form of an inflected verb was used); (b) Correct Uninflected (the preamble was repeated correctly and an uninflected verb was used); Agreement Error (the preamble was repeated correctly and an incorrectly inflected verb was used); Other Error (the preamble was incorrectly repeated, and/or the verb was missing, or there was no response).

#### Analyses

Here, and in the following experiments, analyses of variance were performed on the error data, one with subjects (F1) and one with items (F2) as the random variable.

In addition, statistical analyses were performed by fitting a linear mixed-effects model to error scores using logit mixed-effects models (Jaeger, 2008). We used the lme4 and lmerTest packages in R (version 3.2.3; CRAN project; R Development Core Team, 2008). Included in each analysis were by-subject and by-item random intercepts, and, if warranted (i.e., if the random intercepts analysis showed a significant effect), also random slopes. The models contained as fixed and random effects the same factors as in the analyses of variance.

We provide the results of the ANOVAs for each experiment, with a brief reference to the results of the mixed-effects modeling with further details of these results offered in the endnotes.

### Results and discussion

The results are shown in Table 1.

**Table 1:** **Results of Experiment 1 (Auditory Preambles)**.

**Type**	**Examples**	**Agreement error (%)**	**Correct inflect-ED (%)**	**Correct uninflected (%)**	**Other errors (%)**
sSs	The elf's house with the tiny window	1	67	22	10
sSp	The elf's house with the tiny **windows**	8	49	19	24
pSs	The **elves**' house with the tiny window	2	57	20	21
pSp	The **elves**' house with the tiny **windows**	12	43	17	28

Percentage of responses in each response category for each preamble type. Preamble examples are coded by type (s = singular, p = plural, uppercase = head). Within preamble examples, the head is underlined, and plurals are boldfaced. (Note: due to rounding error, rows sum to 100% only approximately).

More errors were associated with plural local nouns than singular ones. Analyses of variance revealed this to be significant. *F*1_(1, 43)_ = 29.900, *p* < 0.001; *F*2_(1, 19)_ = 19.106, *p* < 0.001. The effect of possessor number was not significant [*F*1_(1, 43)_ = 2.342, *p* = 0.113; *F*2_(1, 19)_ = 2.021, *p* = 0.171, and the interaction of the two variables was not significant (p's > 0.33]. Mixed effects modeling showed the same pattern: only the main effect of local noun number was significant^3^.

A pairwise test of the two conditions containing only one plural [plural possessor (pSs) and plural local noun (sSp)] revealed a significant difference [*F*1_(1, 43)_ = 9.38, *p* = 0.002; *F*2_(1, 19)_ = 9.73, *p* = 0.002]. The mixed effects analysis also showed a significant difference^4^.

Data for the other response conditions were not analyzed statistically; they are displayed in order to show that for the two preamble types in which a single element is plural (pSs vs. sSp), the “opportunity” for an agreement error (derived by summing agreement errors and correctly inflected verbs) is similar, and that the Correct Uninflected and Other Errors are similar in magnitude.

Although possessor number had no statistically significant effect on error production, we note that, numerically, more errors were associated with the plural possessor items than the singular possessor items. This difference could become statistically significant with greater power and a more challenging task. This is the motivation for Experiment 2.

## Experiment 2—visually-presented preambles, possessor modifies head

In this experiment, we used a visual mode of presentation of stimuli in order to increase the overall error rate. Past experiments from our lab have indicated that visual presentation typically results in more errors than auditory presentation. Visual presentation also allowed us to use orthographically distinct cases such as *girl's* vs. *girls'* so that we could increase the number of preambles. Finally, in order to further increase the production of usable data, we included an adjectival ending to promote the use of the copula, which is inflected for number.

### Method

#### Participants

There were 40 participants in this experiment, drawn from the same population as Experiment 1.

#### Materials and procedure

Each preamble was paired with an adjective that participants would be asked to use in their sentence completions. We used the 20 quadruplets used in Experiment 1 and created 20 additional quadruplets, for a total of 32. These were counterbalanced across four presentation lists, as described above. Each list also contained 56 filler preambles. Twenty-four of these contained a plural head modified by a singular or plural possessor and by PP containing a singular or plural head. There were also 32 preambles containing a head and PP. Of these, 20 contained a plural head and singular or plural local noun and 12 contained a singular head and singular or plural local noun. Across the set of 88 items, half contained a singular head and half contained a plural head. The preambles were presented in a different random order to each subject, but always preceded by 8 practice trials.

During the experiment, the preamble appeared along with an adjective, as follows: *The elf's house with the tiny window…cute*. Each preamble appeared for approximately 2 s. Participants were asked to silently read each preamble and adjective and then say a complete sentence out loud. They pressed a foot-pedal to advance to the next item.

#### Scoring and analyses

The same response categories and statistical analyses described previously were used here.

### Results and discussion

The results appear in Table 2. As the table shows, the error rates were indeed higher than in Experiment 1.

**Table 2:** **Results of Experiment 2 (Visual Preambles)**.

**Type**	**Examples**	**Agreement error (%)**	**Correct inflect-ED (%)**	**Correct uninflected (%)**	**Other errors (%)**
sSs	The elf's house with the tiny window…cute	4	95	0	2.0
sSp	The elf's house with the tiny **windows**…cute	28	69	0.6	1.0
pSs	The **elves**' house with the tiny window…cute	11	88	0	0.2
pSp	The **elves**' house with the tiny **windows**…cute	35	63	0.3	0.3

Percentages of responses in each category, for each preamble type.

Analyses of variance revealed a significant effect of possessor number [*F*1_(1, 39)_ = 10.75; *p* = 0.002; *F*2_(1, 31)_ = 28.63, *p* < 0.001], and a robust effect of local noun number [*F*1_(1, 39)_ = 66.62, *p* < 0.001; *F*2_(1, 31)_ = 88.07, *p* < 0.001]. The two factors did not significantly interact (p's = 1.0). Mixed effects modeling showed significant main effects, but also a marginal interaction^5^.

A comparison of the sSp condition (e.g., *The elf's house with the tiny windows*…) with the pSs condition (e.g., *The elves' house with the tiny window*…) reveals a significant difference between the two, shown by ANOVAs [*F*1_(1, 39)_ = 32.94; *p* < 0.001; *F*2_(1, 31)_ = 41.59; *p* < 0.001] and mixed effect modeling^6^.

Data from the other categories were not analyzed statistically. The percentages of correctly inflected verbs complement the Agreement Error results. Very few uninflected verbs were used, and there were very few errors in the Other category.

These results show that, within this more challenging task, plural possessors can induce attraction errors, though significantly fewer than plural local nouns. Further, the presence of a plural possessor and plural local noun appear to have additive effects, resulting in a relatively high rate of errors in the pSp condition.

The next study investigates whether increasing the distance between a potential attractor and the head reduces the potency of the attractor.

## Experiment 3—visually-presented preambles, possessor modifies local noun

This experiment was conducted in order to explore the effect of a plural possessor when it appeared with the local noun. Just as in the previous experiments, local noun number was also manipulated. Here, it is especially important to show that local nouns induce attraction effects; if the local noun induces errors, it must be co-active with the head, and if it is, then the possessor must also be co-active with the head.

### Method

#### Participants

There were 40 participants in this experiment; again drawn from the same pool as in the previous experiments.

#### Materials and procedure

The materials from Experiment 2 were revised to create sensible preambles containing local nouns modified by possessor phrases. Within each quadruplet, the head noun was always singular, the possessor was either singular or plural, and the local noun was either singular or plural [see the examples in (11)]. The filler items were identical to those used in Experiment 2, except that in the 24 fillers containing possessors, the possessor now appeared with the local noun.

(11)
  - Sss The statue in the elf's garden …amusing.
  - Ssp The statue in the elf's **gardens** …amusing.
  - Sps The statue in the **elves'** garden …amusing.
  - Spp The statue in the **elves' gardens** …amusing.

The procedure was identical to that of Experiment 2.

#### Scoring and analyses

These were identical to those used in Experiments 1 and 2.

### Results

The percentages of agreement errors across conditions appear in Table 3.

**Table 3:** **Results of Experiment 3 (Visual Preambles)**.

**Type**	**Examples**	**Agreement error (%)**	**Correct Inflect-ED (%)**	**Correct uninflected (%)**	**Other errors (%)**
Sss	The statue in the elf's garden…amusing	4	92	2	3
Ssp	The statue in the elf's **gardens**…amusing	23	66	2	10
Sps	The statue in the **elves'** garden…amusing	6	89	1	4
Spp	The statue in the **elves' gardens**…amusing	24	64	1	10

Percentages of responses in each category, for each preamble type.

Statistical analyses of the agreement errors revealed a robust effect of local noun number [*F*1_(1, 39)_ = 79.77, *p* < 0.001; *F*2_(1, 31)_ = 104.52, *p* < 0.001], and a non-significant effect of possessor number [*F*1_(1, 39)_ =.92, *p* = 0.343; *F*2_(1, 31)_ = 1.05, *p* = 0.307]. The interaction of the two variables was not significant (p's = 1.0). A comparison of the conditions in which only one element was plural—Sps vs. Ssp—revealed a significant difference [*F*1_(1, 39)_ = 43.45, *p* < 0.001; *F*2_(1, 31)_ = 40.76, *p* < 0.001]. The effects appear not to be additive. Results of mixed effects modeling showed exactly the same effects^7^.

In contrast to Experiment 2, the appearance of a plural possessor downstream from the head has virtually no effect on the rate of agreement errors. A comparison of error rates across Experiments 2 and 3 for the conditions in which a plural possessor appeared with a singular head and local noun (pSs vs. Sps) revealed a significant difference by both ANOVA [*F*1_(1, 78)_ = 5.6, *p* = 0.02; *F*2_(1, 62)_ = 6.02, 0.017], and mixed-effect analyses^8^.

### Discussion

Overall, our results have shown the following: (a) The closer the possessor attractor to the head noun, the greater the likelihood of verb agreement errors. This cannot be reducible to a processing window effect because the local noun attractor in downstream position does produce errors (showing that it is within the same processing window as the head), providing support for the relative proximity hypothesis. (b) Plural possessors in general induce few errors, suggesting that the cue to non-headedness provided by the possessive ending is robust in the same way overt case-marking is, and quite distinct from the cue that is specified by a preposition, lending support for the noun phrase-internal syntactic analysis of the possessive ending.

This latter result is consistent with findings for case-marking languages like Russian (Nicol and Wilson, 1999; Lorimor et al., 2008), which show low rates of error. But note that some of the research on case-marking languages has shown that phonological distinctiveness also plays a role. For example, Hartsuiker et al. (2003) found that an attractor with unambiguous non-nominative case marking induced fewer errors than a case-ambiguous attractor. We observe that the two variants of the possessive ending, ['s] and ['], differ in salience (both phonological and orthographic), and question whether salience plays a role in the effectiveness with which the possessor ending dampens errors.

In order to assess whether this kind of form-related distinctiveness played a role in our studies, we conducted a *post-hoc* analysis of the data from Experiment 2, the only experiment in which possessor number had a significant effect. We divided the items into two groups: plural possessors which marked the possessive with the morpheme –s (e.g., *policewomen's, children's, councilmen's*, etc…) vs. those which marked the possessive only with an apostrophe (e.g., *companies', families', elves'*). The former set of materials contained 15 items; the latter set 17 items. The mean percentages of agreement errors are displayed in Table 4.

**Table 4:** **Experiment 2 data, grouped by type of case-marking**.

**Case-marker on plural**	**Type**	**Examples**	**Agreement error (%)**
	sSs	The woman's position on the issue	2
**‘s**	sSp	The woman's position on the **issues**	23
	pSs	The **women's** position on the issue	8
(*N* = 15)	pSp	The **women's** position on the **issues**	28
	sSs	The country's response to the attack	5
**‘**	sSp	The country's response to the attacks	33
	pSs	The **countries'** response to the attack	15
(*N* = 17)	pSp	The **countries'** response to the **attacks**	43

Shown here are percentages of responses in each category, for each preamble type and case-marking type.

As Table 4 shows, there were more errors when case-marking was less salient (orthographically and phonologically).

ANOVAs showed a main effect of case-marking type [*F*1_(1, 39)_ = 12.75, *p* = 0.001; *F*2_(1, 30)_ = 6.73, *p* = 0.015]. Type of case-marking did not interact with possessor number. Linear mixed effects analyses showed the same effects^9^.

Overall, then, we have seen that both structural and morphophonological variables affect the rate of agreement errors. But we can flesh out the picture even further by investigating semantic effects.

Research by Pearlmutter and his colleagues (e.g., Solomon and Pearlmutter, 2004; and by Brehm and Bock, 2013) has shown that the extent to which a head and local noun are integrated—in a semantic sense—affects whether the ensuing verb is singular or plural. For example, the component elements *drawing* and *flowers* are more tightly integrated in *the drawing of the flowers* than in *the drawing with the flowers*. Interestingly, although Solomon and Pearlmutter (2004) found more agreement errors associated with preambles of the former type (the “of” preambles) than the latter, Brehm and Bock (2013) found the opposite. Brehm and Bock posit that highly integrated phrases such as *the drawing of the flowers* are simply more likely to be treated as a unitary conceptual object (at what is called the “message level” representation, the conceptual representation that feeds the production system). If such phrases are construed as singular, they will be treated as the unmarked singular in the linguistic representation. In contrast, *the drawing with the flowers* is more likely to be treated at the message level as referring to several objects, and thus would be more likely to be marked linguistically with a plural feature.

In phrases containing PP modifiers, the relationship between the head and local noun is signaled by the preposition. But with possessors, the relationship must be computed based on real-world knowledge. Possessors can serve sometimes as arguments to the head (e.g., bearing the agent role in *the salesman's promise* to *the customers*) but need not. Possessors have a very broad and essentially unlimited range of possible connections to the head noun^10^: *the elf's house* can be the house owned by the elf, occupied by the elf, designed by the elf, in which the elf is kept as a prisoner, where the elf bakes cookies, defended by the elf as a matter of duty, etc…

Do speakers compute these various relationships? To address this question, we divided our materials based on which preposition would be used if the possessor-head relationship were recast as a head-PP relationship, choosing the most appropriate preposition in each case. For example, the *women's position* would be recast as *the position of the women* and the *spokeswomen's announcement* would be recast as *the announcement by the spokeswomen*. The semantic integration/referential subordination notion aligns with the preposition choice in our recasting of our materials. In the cases with high referential subordination, the preposition in the converted materials is *of*, unique among prepositions in having no lexical-semantic meaning (it is, for example, the default preposition used with objects of deverbal nouns: *announce the award, announcement of the award*, where the complement of the verb has no accompanying preposition, and the same thematic role between verb or noun and object is understood). The less integrated, less referentially subordinate possessors tend to be converted with prepositions with lexical meaning: *from, by*, and *to*.

We grouped the “of” versions together (seventeen items), and the other conditions together (fifteen items). Results appear in Table 5.

**Table 5:** **Analysis of Semantic Differences associated with the Possessor (data from Experiment 2)**.

	**Type**	**Agreement error (%)**
Possessive = “Of” (*N* = 17)	sSs	3
	sSp	24
	pSs	8
	pSp	29
Possessive = Other (*N* = 15)	sSs	6
	sSp	34
	pSs	13
	pSp	43

Analyses of variance show a main effect of Encoded-Preposition Type (*of* vs. other): *F*1_(1, 39)_ = 12.71, *p* = 0.001, *F*2_(1, 30)_ = 5.84, *p* = 0.022). This variable did not interact significantly with the other variables (which is similar to the Brehm and Bock, 2013, findings). Results of linear mixed effects modeling were similar^11^.

We found significantly fewer errors associated with the “of” versions, in line with Brehm and Bock's findings (2013).

## General discussion

Our findings can be summarized as follows.

First, a possessor attractor that is closer to the head induces more agreement errors than one that is more distant from the head, even when the more distant attractor is co-active with the head^12^. The difference between the two possessor positions shows that relative structural proximity to the head noun is a key factor in determining the magnitude of errors, supporting the view of Vigliocco and Nicol (1998), and arguing against a processing window analysis as an alternative to a proximity account (Nicol, 1995).

Second, compared to a local noun attractor within a modifying PP, plural possessors are much less robust as attractors. Averaging across Experiments 2 and 3, and using the all-singular condition as a baseline, net rates of attraction (subtracting out errors associated with the all-singular condition) were roughly 21.5% for plural local nouns and 4.5% for plural possessors. We have suggested that one reason possessors induce fewer errors is that they carry *within their form* information that they are nonheads. In contrast, when a local noun is the object of a preposition, information about its nonhead status derives from information that is not inherent in its noun phrase: its position within the complex subject noun phrase structure, and the fact that it is the object of a preposition. We conclude that the possessor ending is an element of the noun phrase itself, on a par with case markers, thus supporting the syntactic analysis shown in Figure 2, and arguing against the noun phrase-external analysis of the ending as a separate determiner head (Figure 3).

The fact that form information matters is supported by our first *post-hoc* analysis that showed that the more salient the orthographic/phonological cues about the possessor's role, the fewer errors there were, with possessors with more salient marking such as *women's* causing fewer errors than those with less salient marking like *countries'*.

It is interesting to note that the attraction effect elicited by a possessor as a satellite of the local noun induces fewer errors than that caused by a pronoun in local noun position. Recall the study by Nicol and Antón-Méndez (2009). They showed that a case-marked pronoun in local noun position elicited 6.5% verb agreement errors (5.8% if singular-pronoun errors are subtracted, the *net effect*). This is still substantially larger than the 2% net effect observed in Experiment 3. Obviously, cross-experiment comparison must be interpreted with caution. However, possessors and pronouns are both case-marked, and in the relevant experiments, both intervened between and head and the verb and are roughly the same distance from the root node. In addition, the contrast between singular vs. plural and nominative vs. accusative forms (e.g., *he/him* vs. *they/them*) is more salient than the contrasts in the experiments here. If salience reduces errors, it is even more surprising that pronouns are relatively more powerful attractors. We conjectured that this may be tied to the message level representations of pronouns vs. possessives, specifically with respect to the degree of semantic integration involved.

Our results suggest that the semantic integration between the head and its possessor also matters: when the possessor merely possesses (as in *the elves' house*), fewer errors result than when the possessor is a creator or recipient (e.g., *the congressmen's telegram*). One way in which integration can be understood is that in cases of high integration one entity (the referent of the head noun) is referentially dominant and foregrounded, with the other(s) subordinate to it; this will encourage a singular construal of cases like *the drawing of the flowers*. This referential subordination is reflected in one's intuitions about whether both entities are called to mind with more equal foregrounding. In the case of our possessives, *the elves' house*, plausibly gives rise to a house-dominant conceptual representation, while *the congressmen's telegram* could elicit a representation in which congressmen and telegram are both highlighted (perhaps reflecting the fact that the specifics of a telegram are dependent upon the type of author).

This set of results is consistent with the dominant theory of how verb agreement is computed during language production: the Marking and Morphing model proposed by Bock and colleagues (e.g., Bock et al., 2001; Eberhard et al., 2005). This model assumes a multi-staged architecture in which processing proceeds from top to bottom. First, a non-linguistic proposition (the *message*) leads to the selection of abstract (non-phonological) lexical representations that correspond to concepts within the message, and simultaneously to the computation of a predicate-argument structure. Within the message representation, the roles of the participants are identified, and this information is transmitted to, and coded within, the predicate-argument structure. This includes information about whether, for example, the subject as a whole is singular or plural, and whether the elements that comprise the subject (like modifier-contained noun phrases) are singular or plural. In addition, components of the predicate-argument structure are linked to the abstract lexical representations such that a given lexical item may be assigned to a theme/object role, etc.…At a second stage, a phrasal structure is computed; this structure inherits grammatical number features from the predicate-argument structure. (Other grammatical features are inherited as well, including definiteness, verb tense, and so forth). Verb number is specified via a copying operation that copies number marking from the subject phrase to the verb. Ultimately, form information associated with the selected lexical items is retrieved and slotted into position within the phrasal structure, and inflectional and other grammatical elements are also phonologically realized.

There are two ways for an agreement error to arise. One is during the *marking* process, in which a subject phrase is marked as singular or plural based on its conceptual representation within the message (see also, Vigliocco and Franck's, 1999 *Maximal Input Hypothesis*). Semantic integration of a complex subject exerts its influence here. Following our discussion above, a conceptual level representation corresponding to *The elves' house* will likely be determined to be singular (referring to a singular entity), and marked as such. By comparison, *The congressmen's telegram* will slightly more often receive plural marking, if the message-level representation highlights both congressmen and a telegram.

The other way an error arises within this model is during the later *morphing* process. Morphing involves a set of operations that include connecting lexical information to positions within a syntactic frame that is annotated for number (and other grammatical features), and copying the number feature from the subject noun phrase to the verb (or inflectional node). Part of this process also includes the possibility of percolation of the number feature from the head noun to the root node of the subject phrase. Percolation is a way for the number specification of a head noun to modify the number specification of the subject phrase at the root node (this is described as a “reconciliation” process). (This mechanism is necessary to accommodate cases in which notional number and grammatical number diverge, such as *scissors*, a singular entity with plural marking. If *scissors* is the head, the plural feature percolates to the highest node, effectively turning the subject phrase plural, and triggering plural agreement with the verb). Occasionally, a plural feature from the wrong noun can percolate to the subject's root node, leading to a verb agreement error. The more deeply embedded the attractor, the less likely it is that percolation of a feature would be able to overwrite the phrasal feature. Our results are consistent with this: the greater the distance between a plural possessor and head noun, the smaller its impact.

Morphophonological effects to do with case marking also come about during the morphing process. Bock and Middleton (2011) describe the effect of case ambiguity as follows: “A plausible consequence of this ambiguity is a sparse or unstable feature set when such nouns serve as agreement controllers…this would induce competition between the (intended) nominative and (uninvited but consorting) accusative. In turn, competition increases the likelihood of attraction, which arises when the morphological specifications of an attractor occupy the feature set of the controller.” (p. 1052). In our preambles, the head noun was always case-ambiguous, and therefore subject to competition from the other two nouns, the local noun and the possessor. The local noun was also case-ambiguous, offering greater competition with the head than the case-marked possessor. But in order for case-marking to be useful, it needs to be noticed; our *post-hoc* analysis show that within the set of case-marked possessors, more salient phonological/orthographic case-marking was associated with fewer errors.

## Conclusion

The present results extend the empirical domain of studies of the production of verb agreement by examining possessors, previously unstudied. We have experimentally investigated the magnitude of errors induced by possessors in two positions differing in structural proximity to the head noun, both in comparison to one another and to local nouns in the canonical position investigated in much previous research. We have shown that the higher possessor produces errors at a greater magnitude than the lower, and that both types induce fewer errors than a local noun. These results show that proximity to the head noun matters, and further that some property of possessors significantly dampens errors with this type of phrase, a property we have identified as case marking.

These results bear on three theoretical issues in the account of agreement production. The first is the nature of the proximity effect, where we have argued from the asymmetry between head-modifying and local-noun modifying possessors that relative structural proximity to the head noun plays a key role. The second issue is the role that the possessor ending has in modulating errors, where we have argued it plays a role akin to case marking in richly inflected languages, thus showing English speakers attend to case in spite of the relative lack of case in that language. We have also noted that the salience of the two variants of this ending affects the magnitude of errors, as does the semantic integration of the possessor with the noun it modifies. Finally, we have argued that the psycholinguistic results bear upon the formal syntactic analysis of the possessor ending.

## Author contributions

JN conceived of study, created materials, oversaw lab in which experiments were run, conducted statistical analyses and *post-hoc* analyses, cowrote paper. AB provided linguistic expertise and cowrote paper. JB set up experiments to run, supervised testing of subjects and coding of data, did preliminary statistical analyses.

### Conflict of Interest Statement

The authors declare that the research was conducted in the absence of any commercial or financial relationships that could be construed as a potential conflict of interest.

## Footnotes

^1^All noun phrases have head nouns, but we use the term “head noun” in this article, following common practice in the literature, to refer to the noun that is the structural head of the subject noun phrase, and “local noun” to refer to the head noun of a modifier of the subject head noun. Thus in *the treasure in the cave, treasure* is the head noun and *cave* is the local noun. Throughout the paper, we use the term “noun phrase” to refer to phrases like *the key to the cabinets*, although they are analyzed in some current work as maximal projections of a determiner, i.e., a DP (see Figures 1–4). The DP may be thought of as the “extended projection” of the noun (Grimshaw, 1990). For most of the discussion the distinction between an NP and a DP is not important, and we use “noun phrase” due to its familiarity. We refer to examples like *the elf's house* as a possessive construction, and to *the elf* as the possessor.

^2^It is encoded synthetically on the English pronouns (Nicol and Antón-Méndez, 2009), as a noun affix in Russian (Nicol and Wilson, 1999; Lorimor et al., 2008), and in Slovak (Badecker and Kuminiak, 2007), and on noun-adjacent determiners in German (Hartsuiker et al., 2003). In all these cases the morphological expression of case is structurally within the noun phrase.

^3^Linear mixed effects modeling showed a main effect of Local Noun Number (Estimate = −2.2065, *SE* = 0.4521, *z* = −4.880, *p* = 1.06e-06); the effect of Possessor Number was not significant (*p* = 0.125). The Local Noun Number effect was still significant when by-participant and by-item Local Noun Number random slopes were included (Estimate = 0.08809, *SE* = 0.02499, *t* = −3.525, *p* = 0.001431). A comparison of the models (using X2 tests) showed that inclusion of local noun number random slope provided a better fit to the data. A model that included the interaction of Possessor Number and Local Noun Number showed the interaction to be nonsignificant (*p* = 0.771).

^4^Mixed effects modeling showed a significant effect of Attractor Type (Possessor vs. Local Noun): Estimate = 1.5956, *SE* = 0.5652. *z* = 2.823, *p* = 0.00476. However, with the inclusion of random slopes, the model failed to converge.

^5^The mixed effects analysis of the data from Experiment 2 showed a significant effect of Possessor Number (Estimate = 0.5577, *SE* = 0.1593, *z* = −3.502, *p* = 0.000462); with the inclusion of Possessor Number random slope, this was still significant (Estimate = −0.06875, *SE* = 0.02200, *z* = −3.126, *p* = 0.0035. (This model was no better than the original model). Local Noun Number was also significant (Estimate = −1.9620, *SE* = 0.1842, *z* = −10.652, *p* < 2e-16); and still significant when the model included Local Noun Number random slope (Estimate = −0.24063, *SE* = 0.03573, *t* = −6.734, *p* = 2.31e-08). This model was significantly better than the original. A model that included the interaction of the two variables showed a just significant effect Estimate = −0.7559, *SE* = 0.3845, *z* = −1.966, *p* = 0.049328). Analyses that also included Local Noun Number random slopes showed only a marginal interaction effect (Estimate = −0.7484, *SE* = 0.3953, *z* = −1.893, *p* = 0.058295). The model that included Possessor Number random slopes failed to converge. A comparison of the first two models showed the second to be a better fit.

^6^The pairwise comparison of the plural possessor vs. the plural local noun conditions showed a significant effect (Estimate = 1.2290, *SE* = 0.2244, *z* = 5.478, *p* = 4.31e-08) that held up when random slopes were included (Estimate = 0.17188, *SE* = 0.04011, *t* = 4.285, *p* = 0.000103). A comparison of the two models reveals the second to be better than the first.

^7^Analysis of Experiment 3 data showed a significant effect of only Local Noun Number (Estimate = −1.9738, *SE* = 0.2152, *z* = −9.172, *p* = < 2e-16). Inclusion of Local Noun Number random slopes: Estimate = −0.18750, *SE* = 0.02765, *t* = −6.780, *p* = 5.43e-08). Chi-square analysis shows the latter to better fit the data than the former. The effect of Possessor Number was not significant (*p* = 0.261). Mixed effects modeling that included the interaction of the two variables was not significant (*p* = 0.430). The comparison of the sPs and ssP conditions showed a significant effect (Estimate = 1.7694, *SE* = 0.2994, *z* = 5.911, *p* = 3.41e-09) that was maintained with the addition of random slopes (Estimate = 0.17188, *SE* = 0.04011, *t* = 4.285, *p* = 0.000103). Comparison of the two models indicated the latter to be superior.

^8^The effect of the plural possessor in different positions within the complex noun phrase subject was analyzed. Linear mixed effect modeling revealed a significant effect (Estimate = −0.7751, *SE* = 0.3630, *z* = −2.135, *p* = 0.0327) that remained significant with the inclusion of random slopes (Estimate = −0.05304, *SE* = 0.02526, *t* = −2.100, *p* = 0.0398). A chi-square test showed the latter model to better fit the data.

^9^Mixed modeling analysis of Case-marking Type: Estimate = 0.6197, *SE* = 0.2331, *z* = 2.659, *p* = 0.00784. With Case-marking Type random slopes: Estimate = 0.07588, *SE* = 0.03055, *t* = 2.484, *p* = 0.018964).

^10^Some nouns have an inherent argument structure, including relational nouns like *sister, friend*, and *mother*, and deverbal nouns like *teacher, author*. With such nouns the dominant reading of the Possessor is that of one of the arguments of the noun, though other readings are available—*the teacher's mother* could be a mother assigned as helper to the teacher, for example. For nouns with no argument structure, the possible semantic connections between the Possessor and noun is unlimited. Partee and Borschev (2003) present the example *John's team*, and observe that it may be the team John owns, founded, works for, is a teammate on, covers as a reporter, is a fan of, runs in a fantasy league, etc. See Partee and Borschev (2003) and Barker (1991) for much discussion.

^11^Possessor semantics (preposition type): Linear mixed effects modeling showed a significant effect of possessor semantics (*of* vs. other): Estimate = 0.6113, *SE* = 0.2338, *z* = 2.615, *p* = 0.008925. A model that included the interaction of the variables showed a just-significant interaction of possessor number and possessor semantics: Estimate = 0.618546, *SE* = 0.309129, *z* = 2.001, *p* = 0.0454. No other interactions were significant.

^12^As shown by the robustness of errors with the local nouns that occur downstream from the possessors, showing them both to be within the same activation window as the subject head noun.
