# Supplementary material for: Minimal Interference from Possessor Phrases in the Production of Subject-Verb Agreement
Source: Front Psychol. 2016 May 2;7:548. doi: 10.3389/fpsyg.2016.00548 (PMC4852295; doi:10.3389/fpsyg.2016.00548)
Supplement: Supplementary file 1 [file ExperimentalStimuli.DOCX]

**Stimulus Materials (all-singular versions)**

**Experiment 1 (Auditory)**

| The saleswoman's letter to the boss |
| --- |
| The congresswoman's critique of the proposal |
| The elf's treasure in the cave |
| The dwarf's gold in the chest |
| The woman's subscription to the newsletter |
| The thief's confession about the accomplice |
| The child's trunk containing the toy |
| The wolf's attack on the pup |
| The midwife's assistant at the clinic |
| The person's reaction to the story |
| The congresswoman's telegram to the lobbyist |
| The saleswoman's decision about the pay raise |
| The elf's journey to the mountain |
| The dwarf's story about the quest |
| The woman's memo to the lawyer |
| The thief's opinion of the punishment |
| The child's story about the unicorn |
| The wolf's trail to the den |
| The midwife's treatment of the patient |
| The person's account of the disappearance |

**Experiment 2 Stimulus Materials (Visual)**

| The fireman's truck with the bright light... visible |
| --- |
| The congressman's telegram to the lobbyist... received |
| The dwarf's message to the old wizard... puzzling |
| The thief's confession of the burglary... confidential |
| The country's response to the attack... immediate |
| The chairman's report to the union... shocking |
| The child's dog with the severe injury... survived |
| The fisherman's agreement with the restaurant... unofficial |
| The elf's house with the tiny window... cute |
| The missionary's plan for the school... unrealistic |
| The salesman's promise to the customer... broken |
| The cleaning lady's job at the apartment... tiring |
| The secretary's memo from the supervisor... harsh |
| The midwife's call to the doctor... urgent |
| The spokeswoman's announcement to the newspaper... astonishing |
| The fairy's hex on the old witch... ineffective |
| The policewoman's car with the loud siren... new |
| The wolf's encounter with the hunter... uneventful |
| The housewife's letter to the representative... hostile |
| The company's concern about the strike... selfish |
| The baby's caretaker in the afternoon... irresponsible |
| The family's problem with the agency... discussed |
| The saleslady's display of the outfit... attractive |
| The policeman's uniform with the blue stripe... starched |
| The child's ball with the red stripe... bouncy |
| The child's kitten with the white paw... adorable |
| The woman's position on the issue... passionate |
| The child's trip to the toy store... costly |
| The enemy's defense against the invasion... weak |
| The laboratory's computer for the experiment... modern |
| The councilman's speech about the tax... convincing |
| The woman's dress with the torn sleeve... inexpensive |

**Experiment 3 Stimulus Materials (Visual)**

| The cake at the senator’s celebration delicious. |
| --- |
| The call to the doctor’s secretary urgent. |
| The computer for the laboratory’s experiment modern. |
| The encounter with the wolf's baby frightening. |
| The plan for the school’s upgrade unrealistic. |
| The problem with the family’s account discussed. |
| The report to the chairman's advisor... shocking. |
| The telegram for the congressman's lobbyist... never received. |
| The announcement from the magazine’s spokeswoman... astonishing. |
| The large dog at the toddler’s party... adorable. |
| The letter to the housewife’s representative... hostile. |
| The message for the wizard’s apprentice... puzzling. |
| The outfit for the saleslady’s display... attractive. |
| The position in the woman's newsletter... unpopular. |
| The promise from the company’s salesman... broken. |
| The speech about the councilman's proposal... convincing. |
| The confession of the thief's accomplice... tape recorded. |
| The disagreement about the corporation's lawsuit... petty. |
| The dress at the designer's showcase... expensive. |
| The hex from the fairy's spellbook... ineffective. |
| The job for the cleaning lady's helper... tiring. |
| The restaurant near the fisherman's dock... closed down. |
| The uniform containing the policeman's key... missing. |
| The visit with the student's teacher... informative. |
| The antique trunk for the child's toy... large. |
| The car with the policeman's insignia... new. |
| The invasion of the enemy's bunker... merciless. |
| The memo from the supervisor's lawyer... harsh. |
| The picnic with the baby's caretaker... enjoyable. |
| The tiny fountain in the elf's garden... amusing. |
| The truck with the fireman's hose... leaving. |
| The response to the country's attack... immediate. |
